# Comparison of Growth of Healthy Term Infants Fed Extensively Hydrolyzed Protein- and Amino Acid-Based Infant Formulas

**DOI:** 10.3390/nu10030289

**Published:** 2018-03-01

**Authors:** Marlene W. Borschel, Geraldine E. Baggs, Jeffery S. Oliver

**Affiliations:** 1 Strategic Research & Development, Abbott Nutrition, Abbott Laboratories, Columbus, OH 43219, USA; 2 Statistical Sciences, Abbott Nutrition Research & Development, Abbott Nutrition, Abbott Laboratories, Columbus, OH 43219, USA; geraldine.baggs@abbott.com (G.E.B.); jeffery.oliver@abbott.com (J.S.O.)

**Keywords:** infant growth, elemental formula, hydrolysate formula, free amino acid-based formula, extensively hydrolyzed protein-based formula

## Abstract

The aim of this narrative review was to assess published growth data for healthy, term, infants consuming extensively hydrolyzed protein-based (EHF), or amino acid-based formulas (AAF). These data may be of use to clinicians managing infants with medical conditions consuming these products. A search was conducted using key terms: amino acid-based, hydrolysate, hydrolyzed, hydrolysed, infant formula, infant formulae or formulas, baby formula, or formulae or formulas, infant, infants, infantile, and growth. Seven controlled, randomized, prospective growth trials of healthy term infants fed EHFs or AAFs at similar time points during the first four months of age met these and other criteria, including that the trial was published in a peer-reviewed journal, subjects were enrolled by ≤14 days of age and were exclusively formula-fed at entry and throughout the duration of the trial, and infants were assessed at regular intervals with weight measures available ideally at 14 days, one, two, three, and four months of age. Results suggested that healthy infants receiving commonly available EHFs and AAFs do not appear to experience accelerated growth as reported for infants fed many standard formulas. Differences in growth patterns were observed with some formulas supporting normative growth patterns during the first four months but others appearing to support markedly lower growth patterns. These observations should be confirmed in well-designed prospective randomized trials. Until that time, it is recommended that EHFs and AAFs be chosen carefully with individual patient needs considered.

## 1. Introduction

Hypoallergenic extensively hydrolyzed protein-based formula (EHF) and amino acid-based formula (AAF) are clinically important formulas in the management of infants with food allergy and various gastrointestinal (GI) conditions. Because clinical conditions necessitating the use of such formulas do not often present immediately after birth and these conditions may influence the digestibility and/or absorption of the nutrients in the formula, it may be difficult to assess the ability of the formula to support the optimal growth of young infants. This may be of particular importance to infants fed these formulas as their growth may have faltered prior to receiving an EHF or AAF. Therefore, assessing the growth of normal term infants fed these nutritionally complete EHFs and AAFs may assist clinicians in selection of the optimal formula for an individual infant.

Growth of exclusively human milk (HM)-fed and formula-fed (FF) infants has been examined in the past and there is agreement that the growth of FF infants differs from that of HM-fed infants [1,2]. In 1995, Dewey et al. [1] compared the growth patterns of HM-fed infants with the World Health Organization/Center for Disease Control (WHO/CDC) growth charts published in 1986 [3]. These WHO/CDC charts were based on the United States (US) National Center for Health Statistics reference data obtained largely from FF infants [4]. When compared to the 1986 WHO/CDC growth charts, HM-fed infants receiving HM for at least 12 months grew more rapidly during the first 2 months of life and for weight, grew less rapidly from 3 to 12 months of age when compared to FF infants [1]. In recent years, WHO developed reference growth charts based on HM-fed infants and these are now the standard growth charts used in many countries [5].

The composition of infant formulas has changed considerably over the past 30 years and it will likely continue to change in years to come as manufacturers continue to try to better mimic the performance of the breastfed infant. When new infant formulas are developed or current formulations are significantly modified, it cannot be assumed that growth of infants will remain unchanged. For example, when earlier growth tables [4] were developed, infant formulas contained significantly more protein than they do today. In the mid-1970’s, standard milk-based formulas available in the US provided a minimum of approximately 2.24 g protein/100 kcal and EHFs provided a minimum of approximately 3.28 g protein/100 kcal. The Infant Formula Act (IFA) in the US continues to require protein content of infant formula to be between 1.80 and 4.50 g/100 kcal since its implementation in 1980 [6]. Currently, standard US milk-based formulas provide a minimum of approximately 2.0 g protein/100 kcal, EHFs provide at least 2.6–2.8 g protein/100 kcal, and AAFs provide at least 2.8–3.1 g protein/100 kcal. In more recent years, research has suggested that higher levels of protein in infant formulas can modify weight gain during infancy [7]. Koletzko et al. [7] examined the rate of weight gain over the first two years of life in infants fed cow milk-based infant and follow-on formula containing either 1.77 and 2.2 g protein/100 kcal or 2.9 and 4.4 g protein/100 kcal during the first 12 months, respectively. Those receiving the lower protein-containing formulas exhibited lower weight gains in the first two years of life and they were more similar to the growth of HM-fed infants than the growth of infants fed the higher protein-containing formulas. Many infant formulas have since been reformulated to decrease the protein content. New regulations were introduced in 2016 in the European Union (EU) to be implemented in foods for special medical purpose (FSMP) formulas by 2020 and state that infant formulas based on cow milk contain 1.8–2.5 g protein/100 kcal and those with a protein source from protein hydrolysates contain 1.86–2.8 g protein/100 kcal [8]. Both types of formulas previously had the maximum allowable protein contents of 3 g/100 kcal. Requirements for protein content of formulas based on free amino acids (FAA) have not been specifically stipulated by the EU.

Growth of healthy term infants fed some EHFs and AAFs has been shown to differ from that of healthy infants fed other formulas or HM [9,10,11]. In 2011, Mennella et al. [10] reported that weight gain of healthy term infants fed a milk-based formula containing 14 g protein/L (2.07 g protein/100 kcal) was accelerated, whereas, weight gain of infants fed an EHF with 19 g protein equivalents/L (2.81 g protein/100 kcal) was normative, closely following the growth of HM-fed infants (WHO) over the first 7.5 months of life.

The aim of this narrative review was to assess published growth data, specifically weight, weight gain, and weight-for-age *z*-score, of healthy, term, FF infants consuming EHFs or AAFs over the first four months of life. In particular, studies that closely followed the US Food and Drug Administration (FDA) recommendations for supporting healthy growth were desired [12]. Differences in growth performance in healthy, term infants fed these EHFs and AAFs may be of use to clinicians managing infants with medical conditions typically consuming these products.

## 2. Materials and Methods

### 2.1. Search Strategy

A literature search was conducted without screening any results by one staff searcher of the Abbott Nutrition Library Resource Center (Columbus, OH, USA) using the key terms amino acid-based, hydrolysate, hydrolyzed, hydrolysed, infant formula, infant formulae or formulas, baby formula or formulae or formulas, infant, infants, infantile, and growth. PubMed and ProQuest Dialog platforms were used to search databases that included Analytical Abstracts, Allied & Complementary Medicine (AMED), BIOSIS Previews^®^, Embase^®^ EMCare, Food Science & Technology Abstracts^®^ (FSTA), Medline^®^, and Toxfile. The search did not restrict languages, publication type, or geography or setting. The search covered the years 1926 to 2017 and the results were screened by one of the authors (M.W.B) and reviewed according to the review eligibility criteria described in Section 2.2.1.

### 2.2. Design

In 1988, the American Academy of Pediatrics (AAP) prepared a report under US FDA contract recommending design of clinical testing of infant formulas for term infants prior to commercial availability [13]. AAP recommended that a feeding-related difference in weight gain more than 3 g/day over the first 3 to 4 months of life when compared to a control was nutritionally significant and that the number of subjects of each gender be sufficient to detect a 3 g/day difference in weight gain (*p* < 0.05) with a power of 0.8 in a one-tailed test (28 infants per gender) [13]. Although not required by law, manufacturers of infant formula available in the US generally submit and publish such data on new formulas. In the decades that followed the original AAP recommendations, more specific details were revealed in proposed drafts of changes to the IFA regarding expectations for growth study designs including age at entry, visit schedule, and the length of follow-up. Thus, the studies included in this review largely met the guidelines suggested in FDA drafts and were similar to those included in the current infant formula requirements published in 2015 [12]. Using the FDA study criteria facilitated comparisons between studies because of similar populations, study design assessment criteria and timing and type of assessments. For most infant formulas, FDA stipulates that the manufacturer of an infant formula “shall demonstrate that the formula supports normal physical growth in infants when fed as a sole source of nutrition by conducting, in accordance with good clinical practice, an adequate and well-controlled growth monitoring study of the infant formula that:

1. is no less than 15 weeks in duration, enrolling infants no more than two weeks old at time of entry into the study;
2. includes the collection and maintenance of data on formula intake and anthropometric measures of physical growth, including body weight, recumbent length, head circumference, average daily weight increment, and average daily recumbent length increment;
3. includes anthropometric measurements made at the beginning and end of the study, and at least four additional measurements made at intermediate time points with three of the six total measurement made within the first four weeks of the study and three measurements made at approximately four-week intervals over the remaining 11 weeks of the study;
4. compares the anthropometric data for the test group to a concurrent control group or groups at each time point and compares the anthropometric data for each infant (body weight for age, body length for age, head circumference for age, and weight for length) in the test group and the control group to the 2009 CDC growth charts, which are incorporated by reference at 106.160; and,
5. compares the data on formula intake of the test group with a concurrent control group or groups and a scientifically appropriate reference” [12].

Manufacturers of infant formulas that are available in the US generally interpret that for nutritionally complete exempt infant formulas, like EHFs and AAFs, that a similar growth study should be conducted to confirm normal physical growth. Exempt infant formulas are indicated and labelled for special use such as formulas for infants born preterm, infants with inborn errors of metabolism, or infants with special nutritional needs, such as those with food protein allergies.

#### 2.2.1. Study Eligibility Criteria

There were multiple eligibility criteria used for inclusion of studies in this narrative review as the goal was to directly compare results of very similar populations at similar ages, for similar outcomes related to growth. These included the following: (1) the trial was a randomized controlled prospective trial in healthy term infants published in a peer-reviewed journal; (2) subjects were enrolled into the trial no later than 14 days of age and were exclusively FF at entry and throughout the duration of the trial; (3) infants were assigned to either an EHF or an AAF; (4) infants were studied from entry until three to four months of age; and, (5) infants were assessed at regular intervals and weight measures were available ideally at 14 days, one, two, three, and four months of age depending on the length of the study.

### 2.3. Study Formulas

Five studies reported data for EHFs [14,15,16,17,18]; four studies reported data for AAFs [14,18,19,20] and the formulas are detailed in Table 1. The protein in EHFs is extensively hydrolyzed, thus, a sizeable percent of the protein is in the form of FAAs, perhaps as high as 50% or more, with the remainder largely in the form of very short peptides. AAFs and EHFs may also contain specialized nutrient sources, such as medium-chain triglycerides (MCT) or structured lipids. Infant formula labeling in the US represents minimum nutrient content/100 kcal. All the formulas reported in this review provided at least 2.6 g protein/100 kcal with some AAFs, providing a minimum of 3.1 g protein/100 kcal (Table 1).

### 2.4. Statistical Methodology

**Figure 1:**
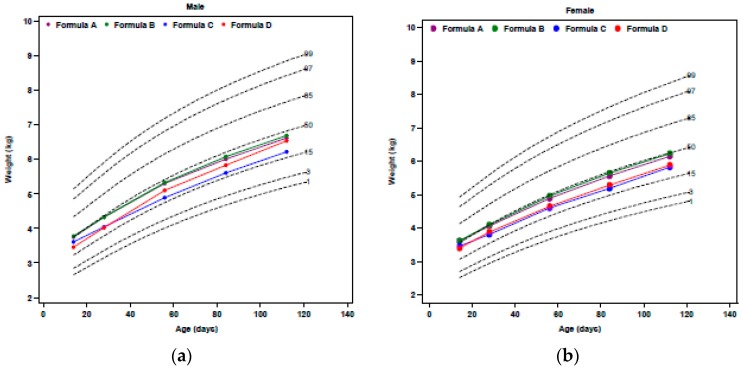
Mean weight-for-age percentiles (World Health Organization (WHO)) of evaluable healthy term infants at 14 day, and one, two, three and four months of age fed extensively hydrolyzed protein-based (EHFs). (**a**) WHO weight-for-age percentiles of male infants; (**b**) WHO weight-for-age percentiles of female infants. Mean weight of infants fed EHF-A and EHF-B were not statistically significantly different during the first four months of life (*p* > 0.05) and were significantly greater than those fed EHF-C and EHF-D at 2 weeks and at 1, 2, 3, and 4 (EHF-C only) months of age (*p* < 0.05). Data source: EHF-A [15]; EHF-B [14]; EHF-C [17]; EHF-D [17].

Most statistical results presented in this narrative review were reported in the specific studies referenced. Additional analyses conducted by AN included the following. Weight (kg) at birth (BW) for the intent-to-treat (ITT) cohort of EHF-fed infants and at each visit for the evaluable subgroup of EHF-fed infants displayed on Figure 1 were compared between groups using two-sided *t*-tests. For EHF-C and EHF-D, sample sizes indicated in the data source consort table [17] were assumed for each visit. Additionally, mean weight gain per day from 14 to 112 or 14 to 120 days of age (Table 2) were analyzed. Separate comparisons were conducted for EHFs and AAFs. *p*-values ≤ 0.05 were considered as statistically significant, without the adjustment for multiplicity of testing. SAS^®^ 9.4 (SAS Institute, Inc, Cary, NC, USA) was used for these analyses.

## 3. Results

Based on the criteria outlined in Section 2.2.1, the literature was reviewed to identify studies that reported the growth of healthy term infants exclusively fed either an EHF or AAF during early infancy. Seven controlled, randomized, prospective growth trials of healthy term infants fed EHFs or AAFs at similar time points during the first four months of life have been reported and are presented in Table 3 [14,15,16,17,18,19,20]. All of these studies, except for one [20], reported data that could be compared at least for some variable to that reported in the other studies. One additional study [10] provided relevant information but the study design failed to meet all the FDA criteria so most data could not be directly compared to that reported in other studies. These studies provided an opportunity to compare growth patterns of healthy, term infants fed these EHFs and AAFs without the confounding effects of clinical conditions, variable ages, and varying study design. All of the studies enrolled exclusively FF healthy term infants that were ≤2 weeks of age at entry. Infant growth was assessed at the enrollment visit (in most studies), at two weeks, and at one, two, three and four months of age.

### 3.1. Compilation of Results of EHF and AAF Growth Studies in Healthy Term FF Infants

#### 3.1.1. Extensively Hydrolyzed Protein-Based Infant Formulas

**Figure 2:**
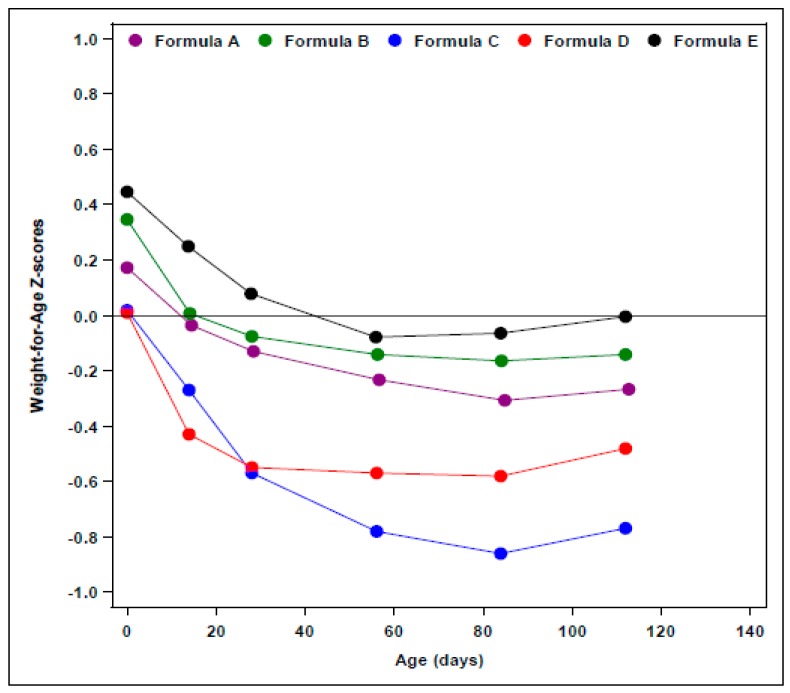
Mean WHO weight-for-age *z*-scores of evaluable healthy term infants fed EHF-A, EHF-B, EHF-C, EHF-D and AAF-E at birth (ITT for EHF-C and EHF-D), 14, 28, 56, 84, and 112 days of age. Data source for EHF-A, EHF-B and AAF-E was from unpublished data from Borschel et al. [14] and Borschel et al. [15] and data source for EHF-C and EHF-D was Fields et al. [17]. An ad hoc analysis of change in mean WHO weight-for-age *z*-score for EHF-A, EHF-B and AAF-E from 14 to 112 days of age showed no statistically significant differences among formulas (*p* = 0.71). Differences in the WHO weight-for-age *z*-scores for EHF-C and EHF-D when compared to EHF-A, EHF-B, and AAF-E could not be determined from the published data.

Results of the five EHF studies assessed growth of ≥52 infants/group [14,15,16,17,18]. WHO weight percentiles [5] of male and female infants fed one of four different EHFs are presented in Figure 1. Data for the powdered form of EHF-A [15] was included in this review although it was consistent with the data for the ready-to-feed form [15]. Data on EHF-B from the remaining studies [16,18] were not included in Figure 1, as, although the data were consistent, they were reported on CDC growth curves [21]. A narrative review of the EHF studies suggested the following:

- Mean BW of infants fed EHF-A, EHF-C, and EHF-D were similar and very close to the 50th percentile [15,17]. Mean BW of infants fed EHF-B was close to the 60th percentile [14]. BW was not displayed in Figure 1.
- Mean weight gains from 14 to 112 or 120 days of age ranged from 27.6 to 29.1 g/day for all EHFs except for EHF-C (25.4 g/day) (Table 2). All of the EHFs appeared to support weight gains of infants from 14 to 112 days of age in the range of HM-fed infants reported by Nelson et al. [22] at 27.5 g/day except for infants fed EHF-C that supported mean weight gains of only 25.4 g/day. For EHFs, using unadjusted comparisons, mean weight gain per day for EHF-C was significantly less (*p* < 0.05) than the mean weight gain per day for each of the other EHFs.
- Mean weight of infants fed EHF-A and EHF-B were not statistically significantly different during the first four months of life (*p* > 0.05) and were significantly greater than those fed EHF-C and EHF-D at 2 weeks and at one, two, three, and four (EHF-C only) months of age (*p* ≤ 0.05) (Figure 1).
- Mean weight of infants fed EHF-A and EHF-B maintained BW percentiles (~50th to 60th percentile) more closely than did infants fed EHF-C and EHF-D.
- Infants fed EHF-C and EHF-D appeared unable to maintain mean BW percentiles of the 50th percentile with mean weight of males dropping as low as the 32nd (EHF-D) and 25th percentile (EHF-C) during the study [17] (Figure 1).
- Fields et al. [17] reported that significantly more ITT healthy term infants fed EHF-C had WHO weight-for-age falling below the 5th percentile than those fed EHF-D. Over the study period, 12 infants (10%) fed EHF-D and 33 infants (22%) fed EHF-C had at least one weight measurement less than the 5th percentile (*p* = 0.011) [17]. For the evaluable cohort, eight infants (12%) fed EHF-D and 17 infants (21%) fed EHF-C had at least one weight measurement less than the 5th percentile (*p* = 0.014) [17].
- WHO weight-for-age *z*-scores were determined in an ad-hoc analysis from two of our reported studies [14,15] and are displayed in Figure 2. No significant differences (*p* = 0.71) were observed in the change in weight-for-age *z*-score from 14 to 112 days of age in infants fed EHF-A (−0.23 ± 0.09), EHF-B (−0.15 ± 0.09), and AAF-E (−0.25 ± 0.10). Exact change in weight-for-age *z*-score for infants fed other EHFs could not be determined as this information was not reported. However, for EHF-C the change in weight-for-age *z*-score appeared to be considerably greater (approximately −0.45 to −0.55 for ITT and −0.30 to −0.40 for evaluable) than those reported for formulas EHF-A, EHF-B, and EHF-D (Figure 2).
- Body mass index (BMI) of healthy term infants fed EHF-A and EHF-B at 14 and 112 days of age was determined in an ad-hoc analysis from two of our reported studies [14,15] and is displayed in Table 4. Mean BMI of infants was 13.9 and 13.7 kg/m^2^ at 14 days of age for EHF-A and EHF-B, respectively, increasing to 16.6 and 16.5 kg/m^2^ at 112 days of age for EHF-A and EHF-B, respectively. BMI data was not reported or could not be calculated for infants receiving EHF-C and EHF-D.

#### 3.1.2. Free Amino Acid-Based Infant Formulas

**Figure 3:**
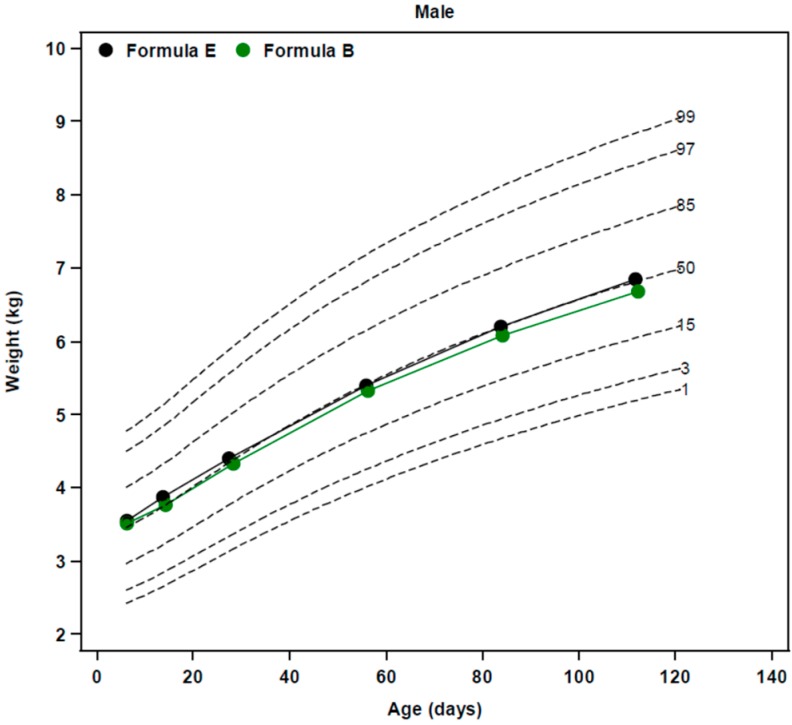
Mean WHO weight-for-age percentiles of evaluable healthy term male infants fed EHF-B and AAF-E to 4 months of age. Data source was from unpublished data from the study of Borschel et al. [14].

Results of three of the four AAF studies [14,18,19] assessed the growth of ≥58 infants/group and the remaining study [20] assessed 38 infants fed AAF-G and 45 infants fed AAF-I. The growth study reported by Harvey et al. [20] failed to report any conventional growth data between 14 to 112 days of age, including mean weight gain per day over the period, mean weights, and lengths at each visit, or a comparison of the weights and lengths to growth standards, such as the WHO reference standards. A narrative review of the AAF study results demonstrated the following:

- BW percentiles of evaluable healthy term male infants fed AAF-E were at the 60th percentile (Figure 3) and tracked very close to the 50th percentile over the first four months of life (ad hoc data obtained from Borschel et al. [14]).
- In contrast, mean weight percentiles of ITT healthy term male infants fed AAF-F were at the 48th percentile at birth and dropped to between the 28th to 36th percentiles (females ranged from 39th to 47th percentile) over the first four months of life [19].
- Similar to infants fed AAF-F, ITT healthy term male infants fed AAF-H had mean weight percentiles at the 50th percentile at birth that dropped to between the 30th to 37th percentiles (females ranged from 31st to 37th percentile) during the study [19].
- Mean weight gains from 14 to 112 or 120 days of age ranged from 27.3 to 29.8 g/day for all AAFs (Table 2), and were similar to those reported by Nelson et al. [22] for breastfed infants (27.5 g/day). Data for AAF-F were taken from Corkins et al. [19] since they were not reported in Harvey et al. [20]. For AAFs, using unadjusted comparisons, mean weight gain per day for AAF-F was significantly less (*p* < 0.05) than mean weight gain per day for AAF-G.
- WHO weight-for-age *z*-score of AAF-E from birth to 112 days of age (Figure 2) and change in WHO weight-for-age *z*-score was determined in an ad-hoc analysis from one of our reported studies [14]. No significant differences were observed in the change in weight-for-age *z*-score from 14 to 112 days of age among infants fed EHF-A, EHF-B, and AAF-E (*p* = 0.71). Healthy term infants fed AAF-E displayed decreases of −0.25 ± 0.10 in weight-for-age *z*-score from 14 to 112 days of age. Weight-for-age *z*-score and change in weight-for-age *z*-score for infants fed other AAFs could not be compared as information was not reported.
- Data for infants fed AAF-G were compared to CDC growth standards and tracked with data for infants fed EHF-B [18]. Mean weights of infants fed AAF-B reported by Borschel et al. [14] tracked closely with those of infants fed AAF-E (Figure 2) and were near the 50th percentile on WHO standards. Therefore, it was assumed that if plotted on WHO growth plots, mean weight of infants fed AAF-G would track close to the 50th percentile.
- Harvey et al. [20] examined infants fed AAF-F (control) when compared to infants fed AAF-I (same formula as AAF-F, but with added pre- and probiotics). Harvey et al. [20] did not detail the protein content of the formulas. AAF-I was commercialized in the US with 2.8 g protein equivalents/100 kcal and the protein content of AAF-F was reduced in the US to this same level shortly before the availability of AAF-I. Thus, it was assumed that both formulas studied by Harvey et al. [20] contained 2.8 g protein equivalents/100 kcal. This was lower in protein content than the AAF-F studied by Corkins et al. [19]. Infant weight comparisons were presented unconventionally as ratios of weight gain (AAF-F/AAF-I = 1.00). Although infants grew similarly on both AAFs, it was impossible to determine how infants grew compared to the other AAFs and to WHO standards as these data, neither percentiles nor *z*-scores for individual groups, were presented. Thus, it was assumed that this group of infants fed AAF-I grew no better than the 30th to 37th percentiles, as reported for AAF-F in the study of Corkins et al. [19].
- BMI of healthy term infants fed AAF-E at 14 and 112 days of age was determined in an ad-hoc analysis from one of our reported studies [14] and is displayed in Table 4. Mean BMI of infants was 14.1 kg/m^2^ at 14 days of age for AAF-E increasing to 16.7 kg/m^2^ at 112 days of age. BMI data was not reported and could not be calculated for infants receiving AAF-F, AAF-G, AAF-H, and AAF-I. Change in BMI of infants fed AAF-E was similar to changes observed in infants fed EHF-A and EHF-B (*p* = 0.42) (Table 4).

## 4. Discussion

The objective of this narrative review was to assess published growth data, specifically weight, weight gain, and weight-for-age *z*-score, for healthy, term, formula-fed infants consuming EHFs and AAFs over the first four months of life. Although EHFs and AAFs are formulated to meet the needs of food allergic infants and infants with other types of GI disorders they are nutritionally complete formulas and should meet the nutritional and growth needs of healthy term infants. Recently, Mennella et al. [10] reported that over the first 7.5 months of life weight gain of healthy term infants fed a milk-based formula was accelerated, whereas, weight gain of infants fed an EHF was normative and closely followed the growth of HM-fed infants [5]. Stringent recommendations for assessment of infant formulas issued by the FDA and the launch or reformulation of several EHFs and AAFs in the US market in recent years has resulted in the availability of a considerable number of relatively homogeneous data sets that could be compared.

During the first four months of life, infant growth is rapid and formula usually provides sole source nutrition if the infant if not breastfed. Thus, assessment of a formula’s ability to support growth is best assessed during this time before solids are introduced. Males grow faster than females during this period of life, therefore, it has been recommended that each gender be studied and sufficient numbers of young infants be enrolled to provide sufficient power to detect a 3 g/day difference in weight gain over a four-month period compared to an appropriate control [13]. Additionally, in the US the FDA recommends that feeding of the formula starts by 14 days of age and growth be assessed at three different timepoints during the first four weeks of the study and then every four weeks thereafter for a minimum of 15 weeks study duration to optimally assess the nutritional adequacy of the formula [12]. This study design can be challenging to accomplish as breast-feeding should be and is the first choice of feeding for virtually all infants.

Current WHO and US growth standards are based on growth of breastfed infants [5]. The rationale for developing new growth standards was largely based on the study of Dewey et al. [1] which confirmed that breastfed infants in Europe and the US displayed different patterns of growth than FF infants [23]. In all of the studies included in the current review, mean birthweights were near or above the 50th percentile for weight as would be expected in a representative population of healthy term infants in the US. Thus, it was surprising that commonly available EHFs and AAFs did not all support growth of these infants closer to the 50th percentile during the first 4 months of life and did not display growth patterns previously reported for FF infants [1]. It has been recently reported [24] that compared to breastfed infants included in the WHO Growth Reference Study [5], infants fed intact whey predominant protein-based formula containing no more than 15.1 g protein/L displayed somewhat lower growth during the first two months of life. After two months of age, growth accelerated matching the growth of breastfed infants at four months of age and then exceeded the growth of breastfed infants from 4 to 12 months of age [23]. This growth pattern for the FF infants studied by Spalinger et al. [24] was similar to that described by Dewey et al. [1].

In the current narrative review, weight of healthy term infants fed some formulas (EHF-A, EHF-B, AAF-E, AAF-G) appeared to track quite closely and in a normative fashion with that of breastfed infants throughout the first 4 months of life, whereas the growth of infants on other formulas (EHF-C, EHF-D, AAF-F, AAF-H, and AAF-I) was markedly different. For the latter group of formulas, the mean weights of infants dropped below the means of breastfed infants by 14 days of age and unlike the formula-fed infants reported by Spalinger et al. [24], remained considerably lower than the breastfed infant and those of infants fed formulas EHF-A, EHF-B, AAF-E, and AAF-G during the first four months of life. It should be noted that although growth for an individual infant may be interpreted as “normal” if the infant tracks between the 25th and 75th percentiles, when means of a larger group of healthy term infants track consistently lower, and more specifically in the 20th and 30th percentiles, some individual infants would be tracking considerably less than this. Indeed, in the study of Fields et al. [17] it was noted that significantly more healthy term infants fed EHF-C (22% of ITT infants) had weight-for-age percentiles at or below the 5th percentile than those fed EHF-D (10% of ITT infants) (*p* = 0.011). This finding would be very unusual and of concern for growth of healthy term infants enrolled in controlled and closely monitored growth trials fed a nutritionally complete infant formula marketed in the US. Indeed, we reviewed the ITT data from our two studies [14,15] to determine the number of infants with at least one weight-for-age observation less than the 5th percentile and found that after 14 days of age, only 1 of 167 infants fed EHF-A (combined powder and ready-to-feed groups), 1 of 72 infants fed EHF-B, and 0 of 67 infants that were fed AAF-E fell below the 5th percentile.

Failure to thrive is a frequently used term for infants with weight-for-age below the 5th percentile [25,26] and a concern for pediatric health care professionals. Thus, although it has been suggested that mean growth of groups of infants fed specific formulas is adequate when above −1 SD of the reference standard [24], it appeared when this standard was used, some individual infants fed EHF-C and EHF-D may have displayed suboptimal growth. In addition to WHO mean weight-for age *z*-scores, mean weight-for-age percentiles were available for infants fed EHF-C. These ranged from the 41st percentile at 14 days of age to the 32nd, 27th, 26th, and 29th percentiles at 28, 56, 84, and 112 days of age, respectively [17]. Weight-for-age *z*-scores were not presented for formulas AAF-F and AAF-H, but mean weight-for-age percentiles reportedly ranged from the 30th to the 37th percentile for AAF-F and the 28th to the 36th percentile for AAF-H over the first 4 months of life [19]. If the lowest percentile means were representative of later ages there may have been concern about the growth of individual infants as there was for some infants fed EHF-C and EHF-D.

We hypothesize that apparent differences in growth of healthy infants fed some EHFs and AAFs are likely related more to the formula than to the infants. Possible reasons for these weight differences might include the following: protein source, availability, amino acid composition, and/or amount; fat source, absorption, and/or amount; amount/bioavailability of key growth nutrients, e.g., zinc; sensory issues affecting intake, other ingredients, or effects of manufacturing or storage stability on nutrient bioavailability.

Although protein source and amino acid profile of EHFs varies, protein source does not appear to account for the differences observed. The overall protein content of all the EHFs was high and relatively similar (at least 2.6 to 2.8 g/100 kcal) and both EHF-A and EHF-B were casein-based hydrolysates and supported normative growth compared to the WHO growth standards over the first 112 days of life, whereas the casein-based hydrolysate EHF-C did not.

AAFs are comprised of mixtures of free amino acids (FAAs). Thus, all of the AAFs reported in this review likely had different FAA blends. It was not possible to compare amounts of individual FAAs or most other nutrients in the formulations studied as this information was not provided but differences in types of amino acids, ratios between various amino acids and levels of various amino acids may influence growth. Of note, all the AAFs provided at least 2.8 to 3.1 g protein equivalents/100 kcal.

Medium-chain-triglycerides, a specialized fat source that is readily absorbed into the portal circulation, have been reported to have a thermogenic effect [27]. However, 33% of fat from MCT did not appear to adversely affect the weights of infants fed EHF-A and AAF-E whereas both EHFs associated with lower weight-for-age percentiles of healthy term infants contained higher levels of MCT (55% of fat for EHF-C, 49% of fat for EHF-D). The effect of increased levels of MCT approaching 50% or more of the fat is unknown but likely contributed to the results observed in the EHF studies reviewed. A small unpublished 16-week growth study was conducted by one of us (M.W.B) in 66 healthy male term infants [28]. Infants were randomized within the first three days of life to receive either a ready-to-feed standard milk-based formula without MCT or a similar formula with either 25% or 50% of the fat as MCT until 112 days of age. Infants that were fed the formula without MCT gained significantly more weight from study day 1 to 56 days of age than did infants fed 50% of the fat from MCT (37 ± 1 g/day vs 32 ± 2 g/day, respectively, *p* < 0.05). Additionally, the caloric efficiency (weight gain in g/100 kcal) of the formula with 50% MCT was approximately 7–8% lower than that of the control formula throughout the study. Caloric content of the formulas was calculated based on analytical results of each formula fed in the study.

EHFs and AAFs are judged by adults to taste worse than standard formulas, thus, sensory characteristics could have influenced infant intake of the formulas. However, infants in all studies reviewed except one [20] appeared to consume sufficient amounts of all the formulas to support growth. Overall, mean intakes in some studies were reported as ranging from 792 mL/day to 882 mL/day [17,19], whereas others reported intakes at each study visit [14,15] with means ranging from 842 to 894 mL/day at 112 days of age. Thus, it seems unlikely that taste influenced any difference in growth patterns among these formulas. For AAF-F, intake data from Corkins et al. [19] was considered more representative (850 ± 332 mL/day) as mean intakes reported by Harvey et al. [20] for AAF-F, even if averaged over the four-month period, were atypically low for healthy young exclusively FF infants during the first four months of life (e.g., 11–12 fl oz/day or 325 to 355 mL/day).

Of interest, daily weight gains were generally lower than those of the FF infants reported by Nelson et al. [22] at the 50th percentile (29.5 g/day). None of the formulas included in this review appeared to support accelerated growth of young infants. Increased protein content of formula is frequently implicated as the cause of accelerated growth but EHFs and AAFs typically have even higher protein contents than standard formulas used from 1965–1987 yet growth appears to be lower when the same amount of protein equivalents are supplied by short peptides or FAAs as compared to intact protein. The exact reasons for this are unknown but some factors that could influence absorption include the base protein, characteristics of the enzymatic digestion used, and the resulting chain length of the peptides [29]. Additionally, Grimble and Silk [30] have reported that the rate of absorption of amino acids was faster and more even from a peptide-based versus free amino acid-based mixture. All of the amino acids may not be transported similarly. The EHFs and AAFs reviewed contained different sources of protein, EHFs had different blends of peptides and FAAs, and AAFs had different blends of amino acids. All of these differences could have modified the utilization of the protein and influenced subsequent growth.

As mentioned previously, allowable levels of protein in most infant formulas have been lowered in recent years in countries outside the US. The implications of reductions in protein content of EHFs and AAFs without sufficient clinical studies in populations requiring such formula are unknown but it is suggested that growth of healthy infants fed reduced protein EHF and AAF be assessed prior to use in intended populations where it may be difficult to assess whether faltering growth is due to the formula or to the infant’s clinical condition.

The degree to which poorly growing patients placed on an EHF or AAF might be impacted by these data obtained in healthy term infants can only be speculated. However, in a study in compromised children [31], AAF-E was substituted isocalorically for the child’s pre-study formula for 80 days. Once receiving formula AAF-E, WHO weight-for-age *z*-scores of children increased significantly over the study period (*p* = 0.0260). Mean weight gains were 7 ± 2 g/day in the 90-days prior to study entry increasing to 19 ± 7 g/day during the study period (*p* = 0.0545) [31]. Of the 19 subjects, the pre-study formula feeding was AAF-F (previous US version without added MCT) in 5, an up-age version of AAF-F in 1, EHF-C in 4, and a modular EHF similar to EHF-C, but with 85% of fat from MCT (3232A, Mead Johnson Nutritionals) in 2. For the remaining seven infants, one each received EHF-A and EHF-B, and five received a complete milk-based nutritional formula. Thus, nearly half of the subjects had been receiving formulas appearing to support lower WHO weight-for-age percentiles from 14 to 112 days of age in healthy term infants.

Limitations of the current narrative review included that it was not a systematic review and may be subject to omission and/or bias. Meta-analytic techniques were not used as formula comparisons were made across studies rather than within studies. Moreover, temporal effects of differences in study timing (earliest study included was conducted in 2000, newest completed in 2013) may potentially be responsible, at least in part, for the observed formula effects. Strengths of this review were that all of the studies were designed in a very similar way, all studies enrolled healthy term exclusively formula-fed infants within the first 14 days of life, all of the studies were conducted in the same country (US), and, for many studies, similar data were reported.

## 5. Conclusions

Results of this narrative review suggest that healthy infants receiving commonly available EHFs and AAFs do not appear to experience accelerated growth despite high protein content, as reported for infants fed some higher protein-containing standard formulas. Some EHFs and AAFs appeared to support normative growth patterns during the first four months of life when compared to reference data for breastfed infants (WHO). Differences in growth patterns of infants fed various EHFs and AAFs were observed. These observations should be confirmed in well-designed prospective randomized trials. Until that time, it is recommended that EHFs and AAFs be chosen carefully with individual patient needs considered.

## Figures and Tables

**Table 1:** Extensively Hydrolyzed Protein- and Amino Acid-Based Formulas Cited in this Review *.

Formula Code	Formula	Manu-Facturer	Formula Type	Protein Source	Protein, g/100 kcal ^†^	Protein, g/L ^†^	% Fat as MCT	MCT, g/100 kcal
A	Similac^®^ Alimentum^®^	AN	EHF	Casein, TYR, CYS, TRP	2.75	18.6	33	1.83
B	Nutramigen^®^	MJ	EHF	Casein ^‡^, TYR, CYS, TRP	2.8	18.9	0	0
C	Pregestimil^®^	MJ	EHF	Casein ^‡^, TYR, CYS, TRP	2.8	18.9	55	3.08
D	Gerber Hypoallergenic HA^™ §^	NE	EHF	Whey	2.6	17.6	49	2.50
E	EleCare^®^ Infant	AN	AAF	FAA	3.1	21.0	33	1.58
F	Neocate^®^ Infant	NU	AAF	FAA	3.1 **	21.0	33	1.49
G	Nutramigen AA^™^ ***	MJ	AAF	FAA	2.8	18.9	0	0
H	Alfamino^™^	NE	AAF	FAA	2.8	18.9	43	2.15
I	Neo-Syn^™^	NU	AAF	FAA	2.8 ^#^	18.9	33	1.49

* All formulas listed are believed to be US formulations which may differ from formulations of the formula in other global markets; ^†^ Minimum label claim per 676 to 680 kcal/L; ^‡^ Formulas B and C have the same hydrolyzed casein source; ^§^ Assumed to be the US formulation of Althéra^®^; ** Current US product contains 2.8 g protein equivalent/100 kcal but 3.1 g protein equivalents/100 kcal version was studied by Corkins et al. [19]; *** No longer marketed in the US. Replaced by PurAmino^™^; ^#^ Formulation launched in the US and assumed to be the formulation studied by Harvey et al. [20]. Abbreviations: AAF, amino acid-based formula; AN, Abbott Nutrition, Columbus, OH, USA; CYS, cystine; FAA, free amino acid; MJ, Mead Johnson Nutritionals, Evansville, IN, USA; MCT, medium-chain triglycerides; NE, Nestlé Infant Nutrition Inc., Florham Park, NJ, USA; NU, Nutricia, SHS International Ltd., Liverpool, UK; TRP, tryptophan; TYR, tyrosine.

**Table 2:** Mean weight gain (g/day) from 14 days of age to 112–120 days of age reported for evaluable healthy term infants from the US fed EHFs and AAFs included in this review.

Formula Code	Type of Formula	Number of Infants	Mean Weight Gain, g/Day	Interval of Weight Gain	Reference
A	EHF	67	27.8 ± 0.7 *	14–112	[15]
B-1	EHF	70	27.6 ± 0.7	14–120	[16]
B-2	EHF	52 ^‡^	29.1 ± 1.0	14–120	[18]
B-3	EHF	69	28.4 ± 0.7	14–112	[14]
C	EHF	58	25.4 ± 6.5 ^†^	14–112	[17]
D	EHF	68	28.1 ± 6.2 ^†^	14–112	[17]
E	AAF	65	28.3 ± 0.8	14–112	[14]
F	AAF	60	27.3 ± 4.9 ^†^	14–112	[19]
G	AAF	58 ^‡^	29.8 ± 1.0	14–120	[18]
H	AAF	59	27.4 ± 6.4 ^†^	14–112	[19]

* Mean ± SEM; ^‡^ Not stated if infants were ITT or evaluable completers; ^†^ Mean ± SD. Abbreviations: AAF, amino acid-based formula; EHF, extensively hydrolyzed protein-based formula.

**Table 3:** Summary of extensively hydrolyzed formula and amino acid-based formula growth studies included in the narrative review.

Study	Formula Code	ITT Infants, Number	Evaluable Infants, Number	Country and Number of Sites	Main Results	Funding Source
Borschel et al., 2014 [15]	EHF-A	95 (54/41) *	67	US, 8 sites	Infants fed liquid and powdered forms of EHF-A had similar daily wt gains. Drop-out rate was 29% on EHF-A.	AN
Scalabrin et al., 2009 [16]	EHF-B	94 (44/50)	70	US, 14 sites	Mean achieved wt for male and female infants fed EHF-B with and without probiotic were similar and plotted on the CDC charts [21] fell between the 25th and 75th percentiles. Drop-out rate was 36% for EHF-B.	MJ
Burks et al., 2008 [18]	EHF-B	165 total enrolled both groups ^†^	52 ^‡^	US, 14 sites	Infants fed EHF-B and AAF-G had similar wt gains. Overall drop-out rate was 33% (percent not reported for each group).	MJ
	AAF-G		58 ^‡^			
Borschel et al., 2013 [14]	EHF-B	106 (55/51)	69	US, 2 sites	Daily wt gain was similar between EHF-B and AAF-E. Drop-out rate was 39% on AAF-E compared to 35% on EHF-B.	AN
	AAF-E	107 (57/50)	65			
Fields et al., 2016 [17]	EHF-C	158 (87/71)	58	US, 25 sites	Daily wt gain was significantly higher on EHF-D compared to EHF-C. Drop-out rate was significantly lower (41%) on EHF-D compared to EHF-C (56%). Infants on EHF-C experienced more days with >3 loose stools/day and a higher incidence of vomiting.	NE
	EHF-D	124 (67/57)	68			
Corkins et al., 2016 [19]	AAF-F	119 (59/60)	60	US, 17 sites	Infants fed AAF-H had similar daily wt gains compared to those fed AAF-F. Drop-out rate was 40% on AAF-F and 47% on AAF-H.	NE
	AAF-H	106 (57/49)	59			
Harvey et al., 2014 [20]	AAF-F	56 (35/21)	38	US, 11 sites	No significant difference in wt gain between AAF-F and AAF-I. Drop-out rate was 46% on AAF-I and 32% on AAF-F.	NU
	AAF-I	59 (35/24)	32			

* Total number of infants (Males/Females); ^†^ Number of ITT subjects not stated for each group; ^‡^ Not stated if infants were ITT or evaluable completers. Abbreviations: AAF, amino acid-based formula; AN, Abbott Nutrition, Abbott Laboratories; EHF, extensively hydrolyzed protein-based formula; MJ, Mead Johnson Nutritionals; NE, Nestlé Infant Nutrition Inc.; NU, Nutricia; wt, weight.

**Table 4:** Mean body mass index (BMI), BMI-for-age WHO *z*-score, and change in BMI-for-age WHO *z*-score from 14 to 112 days of age in evaluable healthy term infants fed formulas EHF-A, EHF-B, and AAF-E.

Formula Code	Type of Formula	Number of Infants	Age, Days	Mean Body Mass Index, kg/m^2^	Mean BMI-for- Age *Z*-Score	Mean Change in BMI-for-Age *Z*-Score from 14–112 Days of Age ^†^	Source of Data for Ad-Hoc Analysis
A	EHF	67	14	13.9 ± 0.13 *	0.16 ± 0.10	-	[15]
		67	112	16.6 ± 0.16	−0.24 ± 0.11	−0.16 ± 0.13	
B	EHF	69	14	13.7 ± 0.12	0.08 ± 0.10	-	[14]
		69	112	16.5 ± 0.14	−0.31 ± 0.09	−0.39 ± 0.12	
E	AAF	65	14	14.1 ± 0.13	0.37 ± 0.09	-	[14]
		65	112	16.7 ± 0.18	−0.15 ± 0.12	−0.41 ± 0.14	

* Mean ± SEM; ^†^ *p* = 0.42 among groups; Abbreviations: AAF, amino acid formula; BMI, body mass index; EHF, extensively hydrolyzed protein-based formula; WHO, World Health Organization.
